# Volumetric Intracardiac Echocardiogram-Guided MitraClip in Patients Intolerant to Transesophageal Echocardiogram: Results From a Multicenter Registry

**DOI:** 10.1016/j.jscai.2023.100594

**Published:** 2023-02-09

**Authors:** Tai H. Pham, Jade Tso, Carlos E. Sanchez, Steven J. Yakubov, Edris A. Aman, Thomas W.R. Smith, Jason H. Rogers, Gagan D. Singh

**Affiliations:** a Division of Cardiovascular Medicine, University of California–Davis, Davis, California; b Division of Cardiovascular Medicine, Riverside Methodist Hospital, Columbus, Ohio

**Keywords:** intracardiac echocardiogram, MitraClip, mitral transcatheter edge-to-edge repair

The success of mitral transcatheter edge-to-edge repair (mTEER) requires high fidelity intraprocedural transesophageal echocardiography (TEE) for optimal transeptal puncture guidance, device steering, and clip implantation. Patients with absolute or relative contraindications to TEE (eg, esophageal/gastric disease, cervical/thoracic spinal disease, and coagulopathies) have limited options. Previous case reports or single-center series have demonstrated the feasibility of volumetric intracardiac echocardiogram (vICE) to provide adjunctive imaging or as a means to guide the entire procedure.^1^^,^^2^ Previously, we highlighted imaging protocol on a limited data set of patients undergoing mTEER with vICE.^2^ In this study, we expand this series and provide safety and feasibility outcomes from a multicenter retrospective series of patients with contraindication or intolerance to TEE undergoing mTEER with the use of only vICE for procedural guidance.

From June 2019 to March 2022, MitraClip cases in which vICE guidance was used were identified, and retrospective baseline demographics, procedural, and follow-up data were collected. Contraindications to TEE were categorized into cervical/thoracic spinal disease, esophageal/gastric disease, and coagulopathies/thrombocytopenia. Procedural success was defined as implantation of at least a single clip with reduction in mitral regurgitation (MR) to 2+ or less. Procedural time was defined as the time from steerable guide catheter (SGC) insertion to clip delivery system removal. Major adverse cardiovascular events, including all-cause mortality, cardiovascular-related death, stroke, and major bleeding (defined as Bleeding Academic Research Consortium life-threatening, disabling, or major bleeding), were analyzed at index hospitalization and 30-day follow-up. vICE guidance was with either the 12.5F 4-dimensional ACUSON AcuNav (Siemens Healthineers) or the 9F VeriSight Pro (Philips). Detailed procedural technique has been described previously.^1^ The vICE system is advanced into the right atrium under fluoroscopic guidance using an appropriately sized sheath from contralateral femoral venous access. For each step, an echocardiologist provides console assistance and image manipulation to help guide the procedure. Then, transseptal puncture is performed in the standard bicaval and short-axis views. The transseptal height is measured using an indirect sweep method because it can be difficult to get a tenting view and mitral valve in the same plane. After puncturing, a 0.025- or 0.035-inch preshaped pigtail guide wire is advanced into the left atrium (LA). Balloon septostomy or catheter floss technique is performed to facilitate crossing of the vICE catheter and MitraClip SGC into the LA.^2^ Then, continuous imaging is used to guide clip delivery system down to the mitral valve. Next, multiplanar reconstruction is used to develop the bicommisural and left ventricular outflow tract, allowing for optimal alignment and trajectory. Entering into the ventricle, grasping, and assessment of leaflet insertion, residual MR location, and severity are performed with similar multiplanar reconstruction views with and without color flow Doppler. After the final clip is deployed and final hemodynamics obtained, the SGC and vICE system are removed.

Baseline demographics of the study population are summarized in Table 1. A total of 12 patients underwent mTEER with vICE guidance. The average age of patients was 71 years, with most being women (n = 7). The mean left ventricular ejection fraction was 53.7% ± 8.6%, with the most of MR being degenerative or mixed etiology (n = 11) and all central in location. The most common contraindication to TEE-guided MitraClip was esophageal/gastric diseases, such as esophageal varices, large hiatal hernias, and esophageal strictures/achalasia (n = 7). The procedure was performed under conscious sedation or monitored anesthesia care in 67% of cases. The catheter floss technique was used in most of the cases (n = 8) to advance the vICE catheter and SGC into the LA. Acute procedural success was achieved in all cases. The average procedural time was 72.8 ± 33.0 minutes. There was a significant reduction in the mean left atrial pressure (before: 16.8 ± 4.6 mm Hg; after: 10.2 ± 5.1 mm Hg; *P* <.05) and left atrial V-wave pressure values (before: 27.7 ± 9.9 mm Hg; after: 17.6 ± 3.7 mm Hg; *P* <.05). There were no cases of leaflet injury or single leaflet device attachment. The average postclip transmitral gradient was 3.3 ± 1.0 mm Hg. The average iatrogenic atrial septic defect at the end of the procedure was 4.8 ± 0.9 mm. In all cases, there was residual left to right shunt. There were no major adverse cardiovascular events during hospitalization or at the 30-day follow-up.

**Table 1:** Baseline demographics of patients undergoing vICE-guided mTEER.

Demographics	n = 12
Age, y	71 ± 17
Male sex	5 (41.6)
Body mass index, kg/m^2^	26.3 ± 3.6
Hypertension	10 (83.3)
Diabetes	3 (25)
Hyperlipidemia	7 (58.3)
Atrial fibrillation	5 (41.6)
Previous CVA	2 (16.7)
Permanent pacemaker	2 (16.7)
Systolic heart failure	2 (16.7)
Contraindication to TEE	
Spinal disease	2 (16.7)
Esophageal/gastric disease	7 (58.3)
Sedation complications	1 (8.3)
Poor TEE windows	2 (16.7)
Baseline TTE	
Left ventricular ejection fraction	53.8 ± 8.5
RVSP, mm Hg	41.3 ± 13.4
Previous valve intervention	0 (0)
MR etiology	
Degenerative	7 (58.3)
Mixed	4 (33.3)
Functional	1 (8.3)
MR location	
Central	12 (100)
Noncentral	0 (0)
MV gradient,^a^ mm Hg	1.8 ± 0.83 (71)
Procedural data	
ICE catheter used	
ACUSON AcuNav	9 (75)
VeriSight Pro	3 (25)
ICE catheter access—contralateral	12 (100)
Successful clip, %	12 (100)
Procedure time, min	72.8 ± 33.05
Fluroscopy time, min	34.8 ± 10.9
Fluoroscopy dose, mGy	134.3 ± 59.8
No. of devices used	
1	8 (75)
2	4 (25)
Device location(s)	
Central	12 (100)
Other	0 (0)
Final device (total used including multiple clips)	
XT/XTR	3
XTW	3
NT/NTR	3
NTW	6
Preclip LA pressure, mm Hg	16.8 ± 3.4
Postclip LA pressure, mm Hg	12.2 ± 2.8
Preclip V waves, mm Hg	29.8 ± 9.2
Postclip V waves, mm Hg	16.8 ± 3.0
4D ICE MitraClip assessment	
Postclip residual MR	
Trace	2 (16.7)
1	6 (50)
1-2+	2 (16.7)
2	2 (16.7)
Postclip mean gradient, mm Hg	3.3 ± 1.0
Leaflet tear	0 (0)
Single leaflet device attachment	0 (0)

Values are given as mean ± SD or n (%).

CVA, cerebral vascular accident; ICE, intracardiac echocardiography; LA, left atrium; MR, mitral regurgitation; MV, mitral valve; NT, narrow short-arm MitraClip; NTW, wide short-arm MitraClip; NTR/XTR, thrid generation narrow short and long-arm MitraClips; PPM, permanent pacemaker; RVSP, right ventricular systolic pressure; TEE, transesophageal echocardiography; TTE, transthoracic echocardiography; XT, narrow long-arm MitraClip; XTW, wide long-arm MitraClip.

a The average heart rate at which the MV gradient was measured and reported was 71 BPM.

In this multicenter registry, over the span of 3 years, the number of patients with absolute contraindication to TEE or with imaging windows insufficient to guide mTEER was small. vICE-guided mTEER was shown to be safe and feasible with a high rate of procedural success in such patients. The procedures were completed without any adverse events related to vICE. The field of vICE-guided structural heart procedures is young but increasing experience and evidence is needed to define its role. This series is small and with inherent limitations, the data suggest that the threshold to use vICE for procedural guidance may be lowered. Most patients presented with either primary degenerative or mixed etiology with central MR although some patients with depressed ejection fraction and secondary MR were treated. Consideration of performing mTEER with monitored anesthesia care sedation and vICE is attractive because general anesthesia for those with advanced cardiomyopathy is not without risk.

Not previously reported, in this multicenter retrospective series, vICE was shown to be a feasible and safe to guide TEER in patients with relative or absolute contraindication for TEE. Further studies are needed to define the procedural role for vICE in these patients.

## Declaration of competing interest

Gagan D. Singh, Jason H Rogers, Carlos Sanchez, and Thomas W.R. Smith are consultants for Abbott Structural Heart, Boston Scientific, and W.L. Gore. Gagan D. Singh, Edris Aman, and Carlos Sanchez are consultants for Phillips and Siemens Healthineers. Tai Pham, Jade Tso, and Steven Yakubov reported no financial interests.
